# Green Thermo-Photo Catalytic Production of Syngas Using Pd/Nb–TiO_2_ Catalysts

**DOI:** 10.1021/acssuschemeng.2c07285

**Published:** 2023-02-20

**Authors:** Uriel Caudillo-Flores, Rocío Sayago, Alejandro Ares-Dorado, Sergio Fuentes-Moyado, Marcos Fernández-García, Anna Kubacka

**Affiliations:** † Centro de Nanociencias y Nanotecnología, Universidad Nacional Autónoma de México, Ensenada 22800, Mexico; ‡ Instituto de Catálisis y Petroleoquímica, CSIC, C/Marie Curie 2, Madrid 28049, Spain

**Keywords:** hydrogen, syngas, thermal and photon dual excitation, methanol, mechanism, in situ spectroscopy

## Abstract

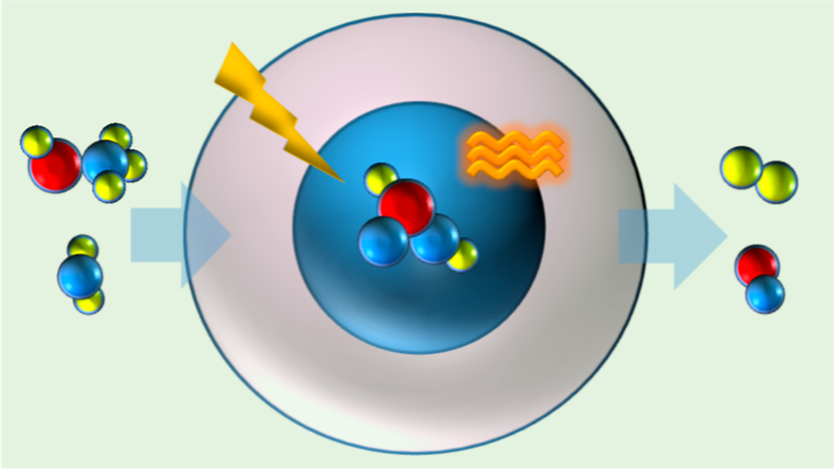

In this contribution, a series of Pd-promoted Nb-doped titania samples were essayed in the gas-phase thermo-photo production of syngas from methanol/water mixtures. The Pd loading was tested in the 0.1 to 2.5 wt % range, leading to the presence of metallic nanoparticles under reaction. Reaction rates exceeding 52 mmol H_2_ g^–1^ h^–1^ and quantum efficiencies above 33% were obtained. The optimum sample having a 0.5 wt % of Pd provided an outstanding synergy between light and heat under reaction conditions, facilitating the boost of activity with respect to the single-source processes and achieving high selectivity to syngas. The spectroscopic analysis of the physico-chemical ground of the activity unveiled that the noble metal interaction with the Nb-doped anatase support triggers a cooperative effect, promoting the evolution of formic acid-type methanol-derived carbon-containing species and rendering a significant enhancement of syngas production. The proposed thermo-photo system is thus a firm candidate to contribute to the new green circular economy.

## Abstract 2

Activation of the sacrificial methanol molecule by Pd/NbTi thermo-photo catalysts renders high and selective production of syngas.

## Introduction

An important research area in chemistry and catalysis concerns the generation of syngas, the mixture of carbon monoxide and hydrogen, from renewable sources. Syngas has received attention due to its possible transformation through easy gas-phase process(es), the relatively low energy consumption of such chemical process(es) and, as a consequence, of its clean utilization as a carbon resource. Syngas founds application in gas-powered engines, the combined production of heat and electricity, but, particularly, in the chemical industry to produce a significant number of building blocks for the chemical and polymer industries.^1−3^ Methanol and, in general, alcohols are products of well-known chemical and biological processes and can be obtained massively using green and environmentally respectful industrial procedures. Moreover, the transformation of methanol with the help of water can be considered a fully renewable and green chemical process for syngas as well as for hydrogen alone production.^1,4−6^

The use of bio-alcohols in the production of hydrogen and (less frequently) syngas has been subjected to numerous studies using bio-alcohols as sacrificial agents. Specifically, the thermal reforming process using water has been tested with numerous catalysts, and well-known reviews have examined the field. The exothermicity of the reaction is a key challenge to carry the reaction at low temperature, typically below 200 °C. The relatively high temperature required for operation of benchmark catalysis based in noble (Pt and Rh) and non-noble (Cu and Ni) supported on reducible oxides as well as the concomitant deactivation effects on the currently available catalysts are issues which do not allow practical implementation of the process at the present stage.^4,7,8^ Alternatively, photo-based activation and transformation of the alcohol can be carried out at room temperature and pressure. However, several physico-chemical phenomena such as the significant charge recombination after illumination or a sluggish kinetics with multiple and successive steps consuming a significant number of charge carrier species limit efficiency and correspond to a major issue for technological application of photocatalysis.^9,10^ Irrespective of the energy source of the methanol-based catalytic process, a combination of light and thermal energy sources can mitigate or eliminate some of the main controlling factors limiting the practical implementation of single-energy source thermal- and photo-alone processes.^11−13^ As a consequence, in recent years, the thermo-photo catalytic production of hydrogen and syngas has been actively investigated.^14−21^ Among tested catalytic powders, TiO_2_-based materials have been broadly used due to the well-known adequate properties of the oxide, both in the thermal and photonic alone processes.^19,22^ Both noble metal^20,23−25^ and non-noble metal^21,26−31^ co-catalysts are customarily used for TiO_2_-based catalysts in order to boost activity. Among them, the study of Pd is scarcely investigated for the production of hydrogen, although has been already tested for volatile organic elimination using thermo-photo catalysis.^32^

In this contribution, we modified the titania reduced oxide by doping. The doping process of the semiconductor has been shown particularly beneficial for the hydrogen photo-production process from sacrificial molecules^16,33−35^ but has been never tested, to the best of our knowledge, for the thermo-photo process. Here, we will show that Nb doping of titania, a doping agent leading to a rather good enhancement factor of photo-activity,^34^ also renders a strong boost of activity under dual excitation using light and heat. To provide evidence of the outstanding activity of the system, the solid(s) performance will be analyzed from several perspectives. The first, and as usually presented in the literature, will focus the global efficiency of the catalytic reaction. Here, we will also analyze in more detail the use of both the thermal and light counterparts of the catalytic process. Moreover, the results show that the Nb doping of the titania and the subsequent effects in the interaction with the noble metal are at the core of the promoting effect exerted on the activity. To interpret such an effect, the reaction mechanism is interrogated using in situ infrared (IR) spectroscopy. This will uncover the chemical basis of the outstanding catalytic properties of the Pd/Nb–TiO_2_ system.

## Experimental Section

### Catalyst Preparation and Characterization

The titania support was prepared using a method to ensure uniform particle size and adequate doping by Nb. A mixture of 46.5 wt % of ethanol (Aldrich), 8.3 wt % of titanium butoxide (Aldrich), an ammonium niobate (V) oxalate hydrate (2.5 mol % of cationic basis of niobium with respect to Ti in the oxide; Aldrich, 99.99%), and 55.3 wt % of deionized water was prepared . The Nb content of the catalysts was selected on the basis of previous studies as well as preliminary tests to optimize activity.^34,36^ The mixture was transferred and heated at 160 °C for 2 min under microwave irradiation (Anton Paar, model Synthos 3000). The suspension obtained was nebulized through a 2 mm nozzle in a YAMATO spray dryer (model DL410), at 2 bars and 200 °C. Drying and calcination (500 °C, 2 h) were used to obtain the doped oxide. A PdNO_3_ (Aldrich) solution was used to introduce the noble metal. To do it, the oxide is suspended in deionized water under agitation. Once the noble metal salt is added to the suspension, a chemical reduction step was carried out with the help of a NaBH_4_ (Aldrich) aqueous solution (Pd/NaBH_4_ molar ratio 1/5). After collection of the solids by centrifugation, drying was carried out at 80 °C. The Pd loadings correspond to 0.1, 0.25, 0.5, 1, and 2.5 wt % on metal basis.

Samples were characterized using inductively coupled plasma optical emission spectroscopy (ICP–OES), Brunauer–Emmett–Teller (BET) and porosity adsorption studies, transmission electron microscopy (TEM), X-ray diffraction (XRD), UV–visible spectroscopy (UV–vis), X-ray photoelectron spectroscopy (XPS), photo-luminescence spectroscopy (PL), and in situ IR spectroscopy. Details can be found in the Supporting Information section.

### Activity Measurements

For activity testing, the catalyst was homogeneously distributed into the inner tube of a gas-phase co-axial thermo-photo reactor depicted in the Supporting Information section. Illumination is obtained by four surrounding lamps while the temperature is achieved with a compensated heating cartridge. The system allows a continuous production of syngas under illumination at the desired temperature and utilizing a methanol/water mixture. The full experimental procedure and analytical detection tools used are detailed in the Supporting Information section (see Figure S1 for a schematic view of the reactor).

The energy efficiency of the reaction can be estimated following procedures established previously. These procedures consider hydrogen as an energy vector.^37,38^ Concretely, eq 1 shows at the numerator the output energy from hydrogen production and in the denominator the input energy of the process.
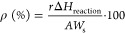
1Here, *r* is the reaction rate (mol s^–1^), Δ*H*_reaction_ is the standard enthalpy of the reaction (237 × 10^3^ J mol^–1^), *W*_s_ is the energy supplied per surface area unit (W m^–2^), and *A* is the surface area of the catalyst subjected to the reaction.

For the reaction rate and quantum efficiency parameters, we also calculated the excess functions with respect to the parent [photo (P) at room temperature and thermal (T) at the corresponding temperature] counterparts. This provides a clear measure of the chemical and photonic effects, leading to a synergistic use of both energy sources. Equation 2 measured the excess reaction rate (*r*_e_).
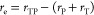
2

Similarly, eq 3 shows the calculation of the quantum efficiency with the help of the normal (*r*) of excess (*r*_e_) reaction rates and the rate of photon absorption (*e*^a^). Both parameters are obtained as average values over the entire volume of the reactor.
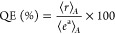
3

Full details of the calculation of the parameters described can be found in refs (12) and (39) and are fully described in the Supporting Information section.

## Results and Discussion

### Catalytic Activity

**Figure 1:**
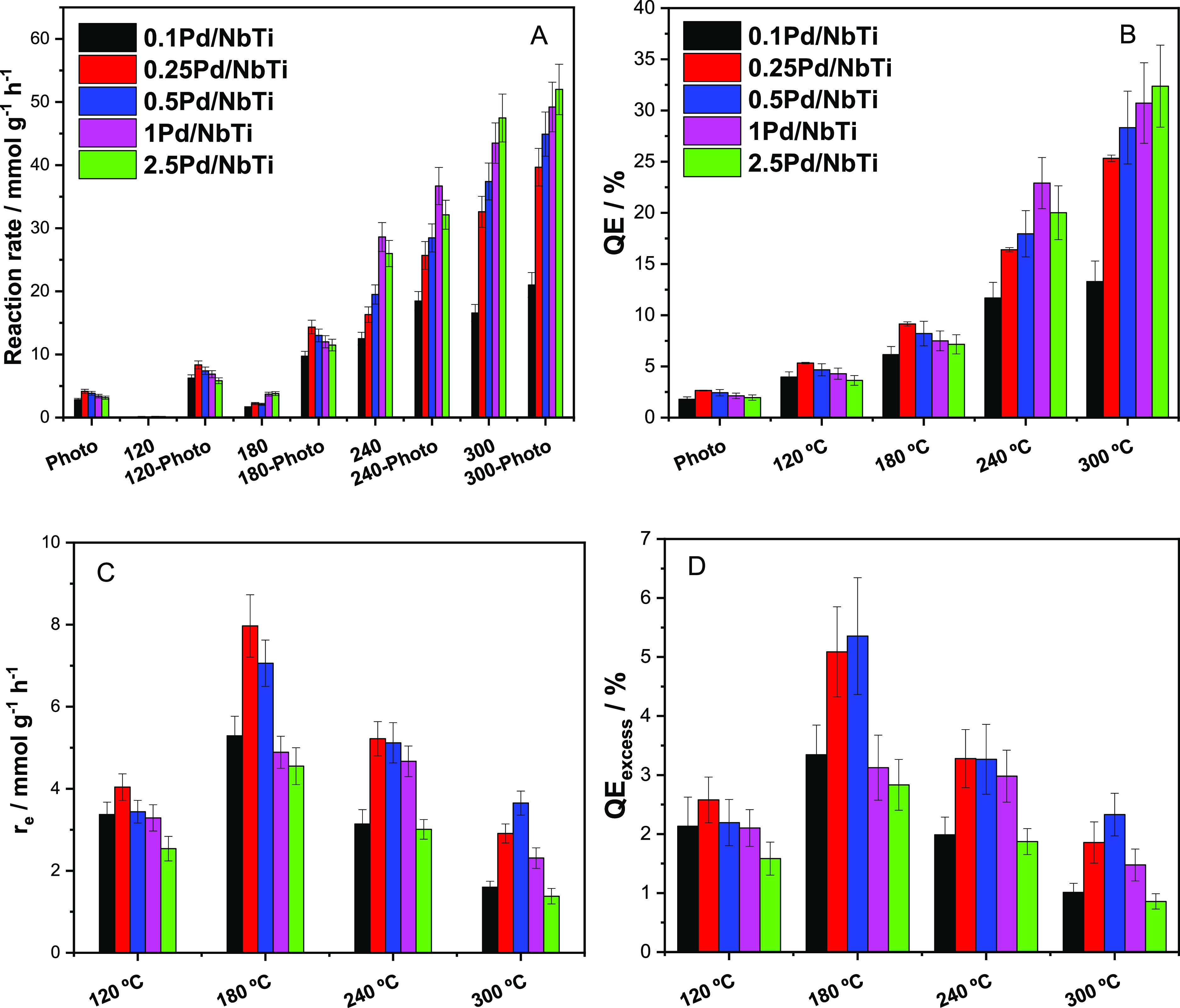
Hydrogen production reaction rate (A) and quantum efficiency (B) as a function of the reaction temperature and sample. Results for photo (room temperature), thermo (at 120, 180, 240, and 300 °C), and thermo-photo (illumination at indicated temperatures) conditions are presented. For both parameters, the excess functions (eqs 2 and 3) are presented at the bottom row panels (C,D).

**Figure 2:**
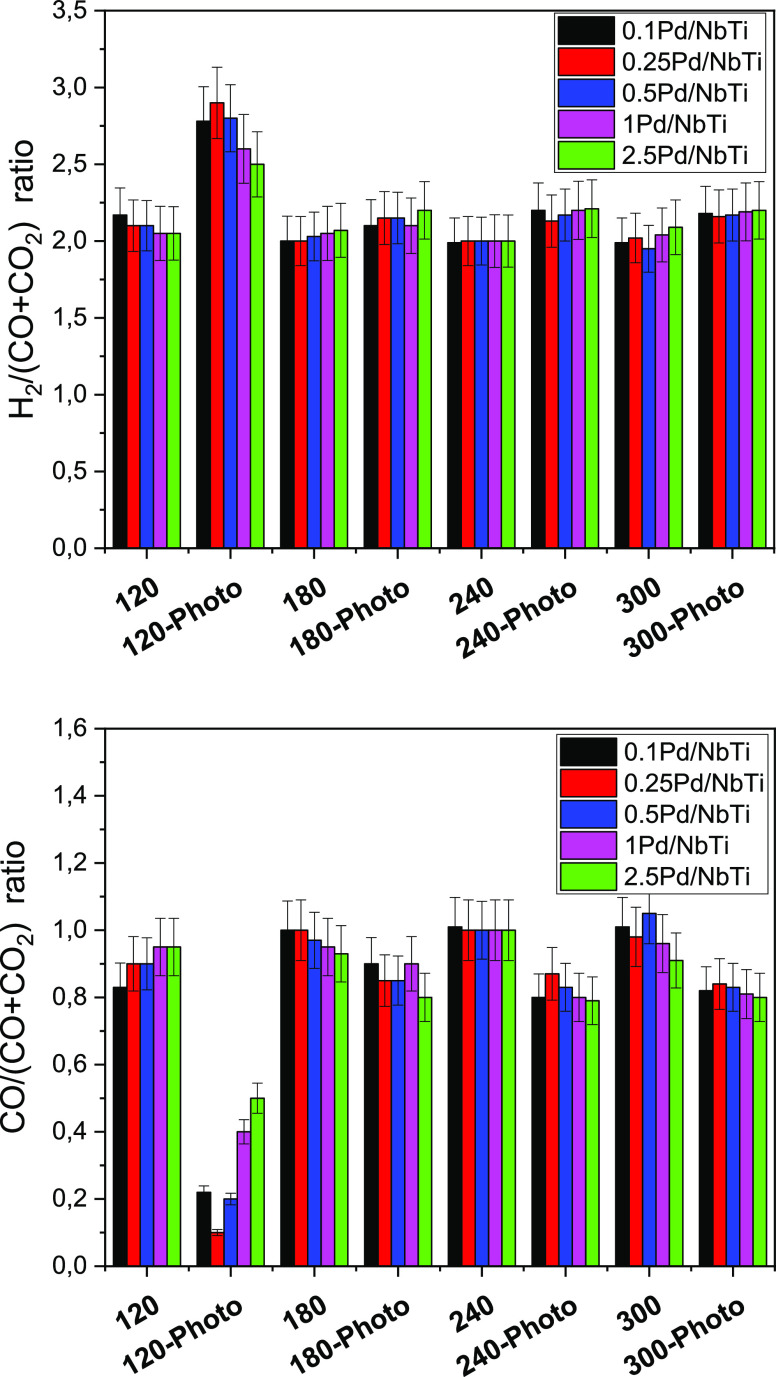
Ratios between hydrogen or CO to carbon oxides as a function of the reaction temperature and sample. Results for thermo (at 120, 180, 240, and 300 °C) and thermo-photo (illumination at indicated temperatures) conditions are presented.

The activity of the samples in the gas-phase reaction is summarized in Figures 1 and 2. To compare with literature reports, hydrogen can be used as a pointing vector of the catalytic process taking place from methanol/water mixtures.^1,4−6^ As shown by both the reaction rate and the quantum efficiency parameters (Figure 1A), the systems are all photo-active at room temperature, irrespective of the Pd content. Photo and (thermo-photo) activity of the bare support is ca. 10 times lower. For all samples, thermal-alone activity is observed above 150 °C. Independently of the Pd content of the sample, the activity grows with the reaction temperature in the whole temperature range explored. There is a fast growth up to ca. 250 °C. Thermo-photo activity is nevertheless observed from 120 °C, a temperature where an essential lack of thermal-alone activity is detected in accordance with literature reports.^4,8^ From Figure 1A, we can also observe that thermo and thermo-photo activity display different behavior versus the noble metal loading. Activity appears to be maximized using intermediate loadings under thermo-photo conditions. Considering the quantum efficiency and restraining to the cases where light is involved as an energy source, all catalysts display an increasing trend with temperature in the quantum efficiency values reported in Figure 1B. The maximum quantum efficiency reaches ca. 25–35% for most of the samples of the series, a range not able to be obtained for any sample under the light-alone excitation.^27,34,40,41^ We would like to stress that for the calculation of the quantum efficiency, all temperature and light-related effects are considered quantitatively, as detailed in the Supporting Information section.^12,39^

Valuable insights concerning the performance of the samples for hydrogen generation can be additionally obtained from the excess parameters (eqs 2 and 3). For the catalysts of the series, the plots at the bottom row of Figure 1 (panels C/D) present the evolution of the excess reaction rate and quantum efficiency parameters as a function of the temperature. Both parameters display similar trends, although they also show subtle differences. As expected, the two excess functions depend on temperature, reaching in all cases the maximum at 180 °C. The excess reaction rate takes maximum values of 55.6 and 54.3% of the corresponding reaction rate of the samples having loadings of 0.25 and 0.50 wt % of Pd. Considering the chemical use of photons at the mentioned temperature, it can be noted that the quantum efficiency parameter for these 0.25Pd/NbTi and 0.5Pd/NbTi samples displays an excess of 55.8 and 65.2%, respectively. Note that the excess function is obtained from the combination of the two sources and not only with respect to the thermal process as the photo-activity contribution is neither negligible nor low. The high efficiency observed using the light and heat combination has been tested under several illumination conditions (UV and visible) for the best sample and optimum temperature (Table S1). Irrespective of the illumination conditions, synergy is observed. To the best of our knowledge, the Pd-promoted Nb-doped titania system appears particularly effective for the reaction, leading to excess function values not observed in the literature.^23,29,42^ At 180 °C, the energy efficiency calculated using eq 1 reaches a value of 1.8% for the 0.5Pd/NbTi sample. This can be compared with previous results, a ZnCu/SiO_2_ catalyst reached a 1.2% energy efficiency under 7.9 suns (temperature ca. 250 °C).^43^ Values well below 1% have been reported for the same reaction but at the liquid phase.^44^ The quantitative analysis carried out highlights that Pd/NbTi samples, but particularly the 0.5Pd/NbTi one, are/is able to handle both thermal and light effects synergistically in the whole range of temperatures explored, including those with practical absence of thermal-alone activity. Therefore, the bottom row plots of Figure 1 provide evidence that the synergy is achieved from ca. 120 to 300 °C and not for a single temperature. The synergy between energy sources of the reaction is additionally demonstrated by the calculation of the activation energy (Figure S2). For the 0.5Pd/NbTi sample, the dual thermo-photo excitation decreases the activation energy. A value of ca. 58 kJ mol^–1^ is obtained for the 0.5Pd/NbTi sample, which optimizes the energy barrier of the methanol reforming reaction by more than 70% with respect to the thermal-alone situation.

In parallel, the gas-phase carbon-containing products of the reaction were analyzed. In Figure 2, we display the H_2_/(CO + CO_2_) ratio behavior through the samples of the series under thermo-photo conditions as well as the thermal counterparts. As can be seen, at lower temperatures there are differences if the solid(s) is (are) excited under combined light and heat or exclusively heat conditions. Such differences are, however, significantly diminished at the temperature displaying the maximum thermo-photo effect (180 °C). The behavior at this temperature is characteristic of the high temperature range explored, from 180 to 300 °C (Figure 2). At such high temperature under thermo-photo conditions, a relatively constant ratio, close to 2.1–2.2, is detected in all samples. Figure 2 also includes information concerning the CO/(CO + CO_2_) ratio. In the mentioned high-temperature range, the dominant generation of CO becomes evident for all samples. Apart from carbon oxides, no other carbon-containing product is detected under reaction conditions.

The behavior of the 0.5Pd/NbTi under reaction conditions is also rather stable. At the temperature where maximum synergy is achieved, Figure S3 of the Supporting Information provides a proof that the continuous production of gas-phase hydrogen and carbon oxides is stable under long time on stream periods, irrespective if the activity test is carried out in a continuous or cycling mode. We also tested a 0.5Pd/Ti reference sample without presence of Nb (and produced using the same experimental procedure). At the mentioned temperature (180 °C), the absence of the doping agent leads to decreasing activity by factors of 2.0 and 2.3, as measured by the reaction rate and quantum efficiency parameters, respectively. Therefore, the outstanding and stable catalytic behavior shown by the 0.5Pd/NbTi sample as well as the synergy achieved under dual excitation, both become evident and will be analyzed in the next sections.

### Physico-Chemical Characterization

**Figure 3:**
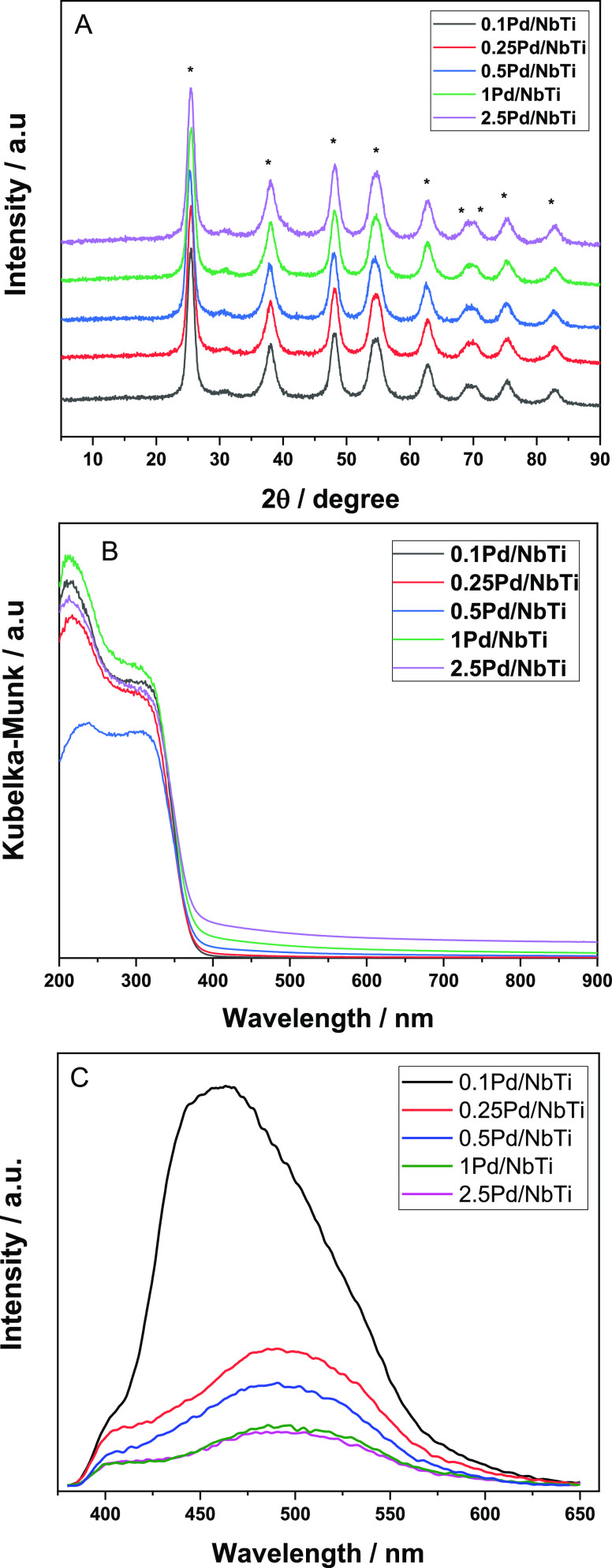
(A) XRD, (B) UV–visible, and (C) PL spectra for the samples. In the XRD plot, an asterisk indicates peaks ascribed to the anatase polymorph.

Figure 3 collects XRD, UV–visible, and PL results for all samples of the series. The XRD patterns reveal the dominant presence of the anatase polymorph (JCPDS card 21-1272; *I*4_1_/*amd* space group) for all samples.^45^ The use of the Scherrer equation showed a nearly constant value of ca. 11–12 nm for the crystallite size of the dominant (by weight) anatase phase present in all samples. The UV–visible spectra appear rather similar for all samples and display a shape characteristic of a semiconductor having a band gap in the UV range, as should happen for anatase. Considering an indirect gap semiconductor, as anatase is reported to be,^46^ the calculation of the band gap energy renders values from 3.06 to 3.20 eV for all samples, with only significant modification taking place for the highest Pd loading. Therefore, a rather modest variation is observed throughout the samples of the series. As the ICP–OES analysis renders a Nb molar content (cation basis) of 2.7 ± 0.3% for all catalysts, a variation of the band gap energy seems to be a mild effect of the noble metal introduction. The main physico-chemical characterization of the catalysts is completed with the morphological analysis of the materials. Table 1 collects values of the BET area and pore volume and size parameters for all samples. In agreement with the more or less constant value of the average particle size obtained from XRD, the BET area of the samples only changes by less than 8%, displaying a modest decrease of the BET area and pore volume with the Pd content. Thus, the physico-chemical (textural) characterization summarized in Table 1 provides evidence of the rather similar support properties along the catalysts of the series. Only the highest Pd loading may occlude some of the pores. The similar physico-chemical properties of the solids may be an expected result considering the preparation procedure and the use of the same support.

**Table 1:** Main Physico-Chemical Properties of the Samples^a^^,^^b^

catalyst	BET surface area (m^2^ g^–1^)	pore volume (cm^3^ gr^–1^)	pore size (nm)	band gap (eV)
0.1Pd/NbTi	129.3/127.6	0.180	5.1	3.20/3.19
0.25Pd/NbTi	125.7/124.9	0.176	5.0	3.18/3.20
0.5Pd/NbTi	124.8/122.6	0.175	5.0	3.15/3.18
1Pd/NbTi	122.9/122.5	0.174	5.0	3.13/3.16
2.5Pd/NbTi	118.6/116.5	0.172	5.1	3.06/3.07

a BET area and band gap parameters are presented for initial (first column) and post-reaction (second column) samples.

b Average standard error: BET area 7.7%; band gap: 0.03 eV.

As mentioned, the ICP–OPES study showed than Nb is present on the materials. The XPS analysis of the materials renders additional information, presenting a rather stable Nb/Ti atomic ratio throughout the series (Table 2). The differences between the average Nb/Ti atomic ratio obtained from XPS and ICP–OES indicate that Nb at ca. 2.5 mol % has no preference or a rather modest preference for being at the surface of the materials. The homogeneous distribution of Nb at the anatase in both pre and post-reaction samples is in agreement with previous studies and indicates that Nb (at this doping concentration) is occupying lattice positions at the anatase polymorph, being thus a true doping agent.^34,47^ For all samples, a Nb 3d_5/2_ XPS binding energy of 209.3 ± 0.1 eV indicates the presence of Nb(IV),^48^ isovalent with the Ti cations of anatase. Note, on the other hand, that the textural and optical properties of solids, mostly associated with the anatase phase, do not suffer significant modification under thermo-photo reaction conditions, as demonstrated by the BET area and pore size/volume values presented in Table 1.

**Table 2:** XPS Ratios for Initial and Post-Reaction Samples^a^

catalyst	Nb/Ti	Pd/Ti
0.25Pd/NbTi	0.031	0.008
0.25Pd/NbTi_Post	0.032	0.008
0.5Pd/NbTi	0.030	0.020
0.5Pd/NbTi_Post	0.029	0.019
1Pd/NbTi	0.030	0.026
1Pd/NbTi_Post	0.031	0.027
2.5Pd/NbTi	0.031	0.094
2.5Pd/NbTi_Post	0.029	0.038

a Average standard error: Nb/Ti 8.9%; Pd/Ti 11.8%.

**Figure 4:**
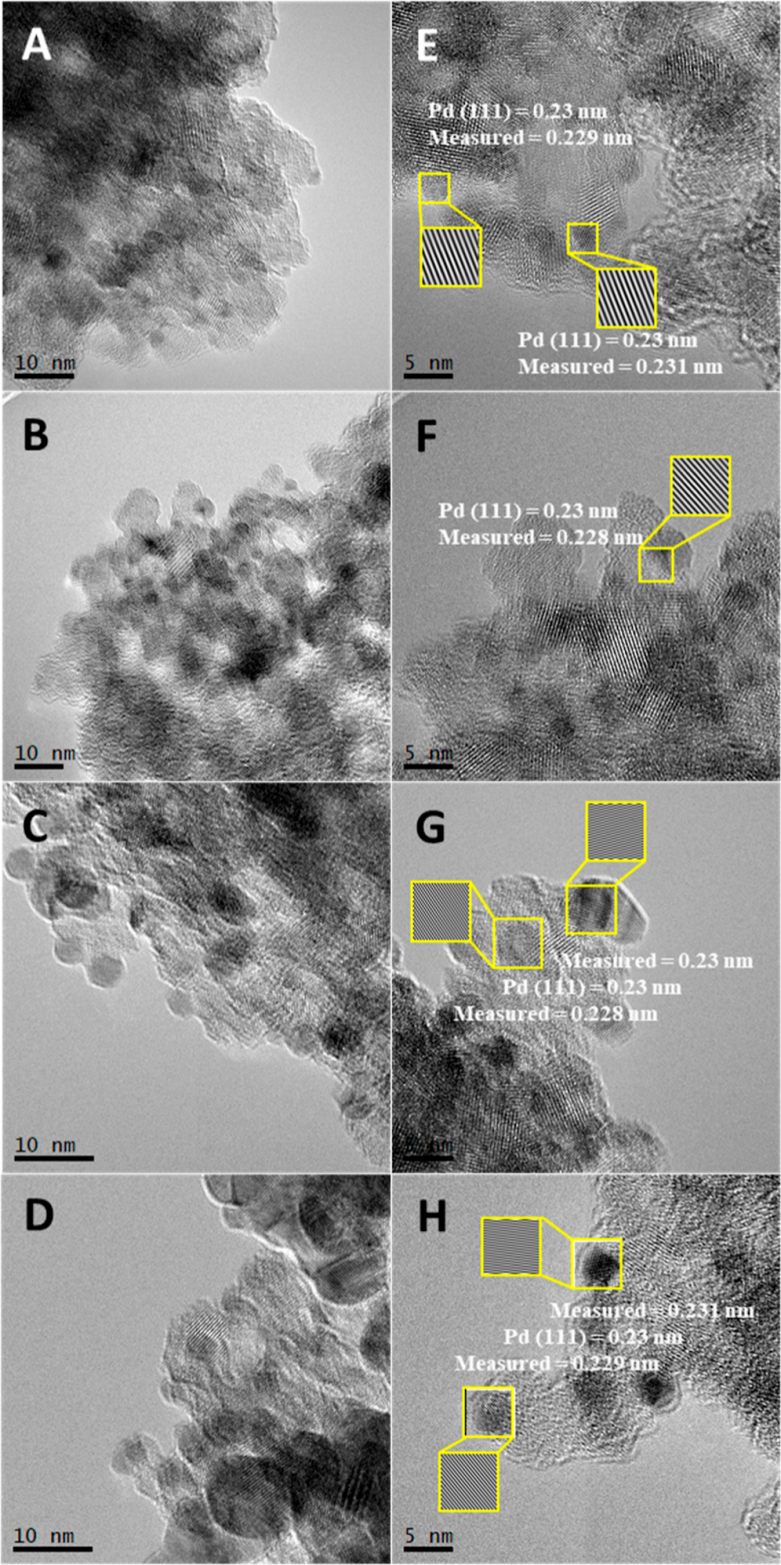
TEM images for the 0.25Pd/NbTi (A,E), 0.5Pd/NbTi (B,F), 1Pd/NbTi (C,G), and 2.5Pd/NbTi (D,H) samples. See the text for details.

**Figure 5:**
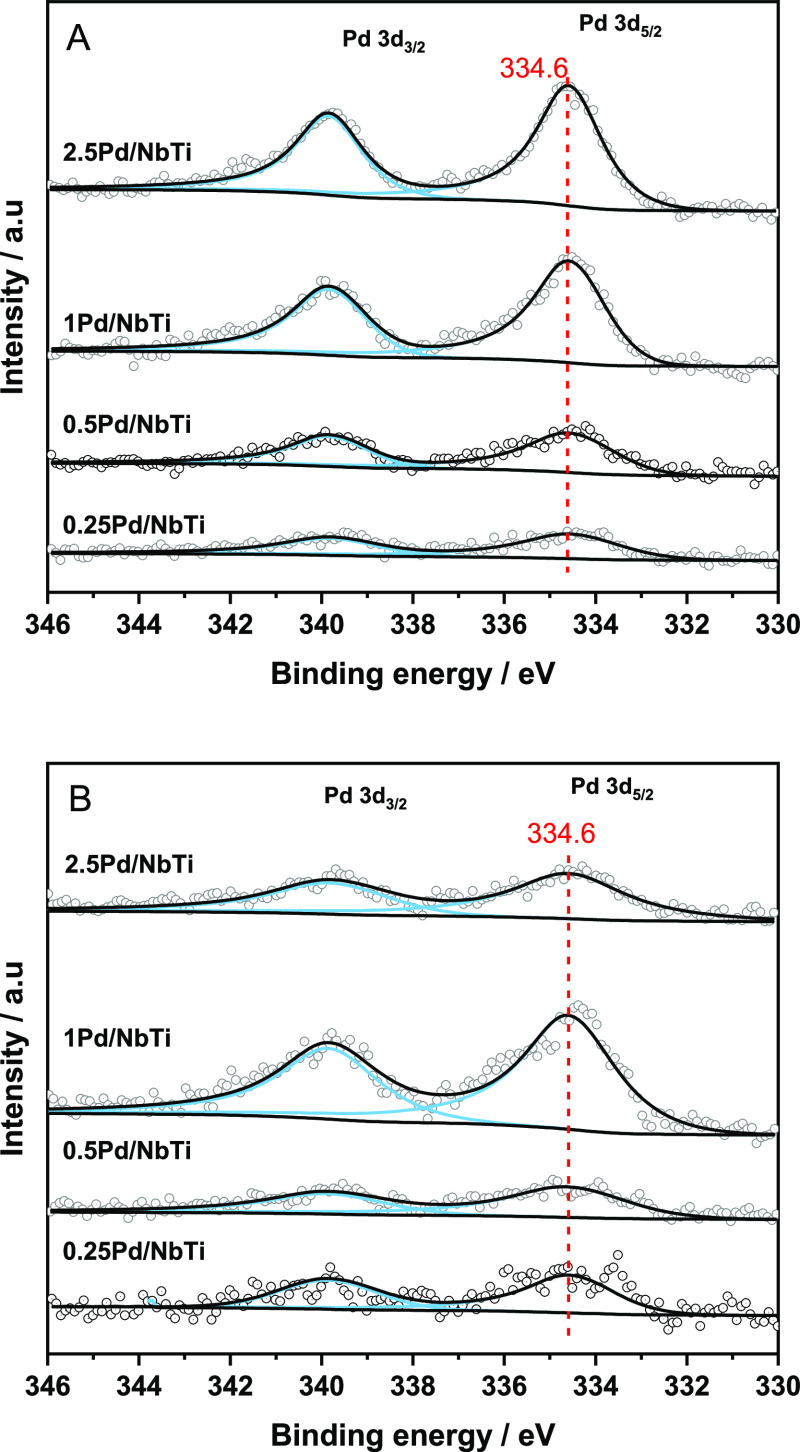
Pd 3d XP spectra of initial (A) and post-reaction (B) samples.

Focalizing now on the study of the noble metal component, information was obtained using microscopy and XPS tools. The TEM images presented in Figure 4 allow us to visualize the round shape of anatase particles with a characteristic dimension of ca. 12 nm, as previously detected using XRD. Over the oxide particles, darker entities corresponding to the noble metal particles are observed. The computed diffracting patterns provide conclusive evidence that the Pd nanoparticles exposed the most stable (111) plane to the surface. Analysis of the Pd particle size distribution for samples having a loading above 0.25 wt % (the 0.1 wt % does not allow us to collect adequate information on the particle size distribution) is presented in Figure S4. This analysis shows an increasing average primary particle size with the Pd loading. Such parameter takes values from ca. 1.9 nm for the 0.25Pd/NbTi sample to 4.4 nm for the high-loading 2.5Pd/NbTi catalyst. XPS was also used to study the Pd component of the solids. Figure 5 depicts results for the Pd 3d XPS region. For all samples, both initial and post-reaction samples give a single contribution peaking at ca. 334.6 eV in all cases. This binding energy is characteristic of the metallic state of the noble metal.^27,48^ The dominant presence of zero-valent Pd nanoparticles is in agreement with the already discussed TEM results. The XPS study also shows that the metallic state is maintained under reaction conditions for all catalysts (Figure 5). The Pd/Ti atomic ratio was also analyzed using XPS, and the values for the catalysts of the series are collected in Table 2. Under reaction conditions, all samples display stability on the values of the Pd/Ti ratio except for the 2.5Pd/NbTi case, where the Pd particle size would grow considerably. Therefore, the XPS analysis was able to show the stability of the Pd-oxidation state and metal particle size under reaction conditions for loadings below or equal to 1 wt %. This fact indicates the stability of the noble metal component and, also, the occurrence of an important metal–support interaction in such Pd-loading regions and can be jointly considered with the previously observed stability of the anatase component of the materials. The characterization data pointed out the stability under reaction conditions of all (noble metal and Nb-doped anatase) components of the samples having a noble metal weight percentage below or equal to 1%. This would justify the stable catalytic behavior presented by the most active 0.5Pd/NbTi sample (see Figure S3).

### Interpretation of Activity

Panel C of Figure 3 shows PL results for the samples under relevant excitation conditions to investigate charge carrier recombination. The response of the samples is characteristic of the dominant anatase phase, presenting the so-called green and red de-excitation peaks, roughly below and above ca. 500–525 nm.^49,50^ PL changes through the series of catalysts correspond to a decrease of the overall intensity together with a shift of the maximum of the signal to higher wavelengths as a function of the noble metal content of the powder. A relevant point concerns the strong effect on the intensity of the signal exerted by the Pd noble metal. This fact indicates, as has been previously discussed in similar systems, that the noble metal decreases significantly charge recombination by capturing electrons and promoting separation of charge after illumination.^27,36^ In the case of our catalysts, we observed a decrease of charge recombination proportional to the Pd content, with a tendency to a leveling off effect for noble metal loadings above 1 wt %. This behavior is in agreement with the already discussed changes on the metal–support interaction throughout the series of samples. Note also that the described shift of the PL maximum would indicate a decrease of surface states associated to the so-called green-type de-excitation as a function of the Pd loading.^27,36^ In any case, although the charge handling and recombination behavior(s) described may have a significant impact on the overall activity trend presented in Figure 1 for the catalysts of the studied series, it does not explain the optimum of the thermo-photo effect, reached with the 0.5Pd/NbTi sample.

**Figure 6:**
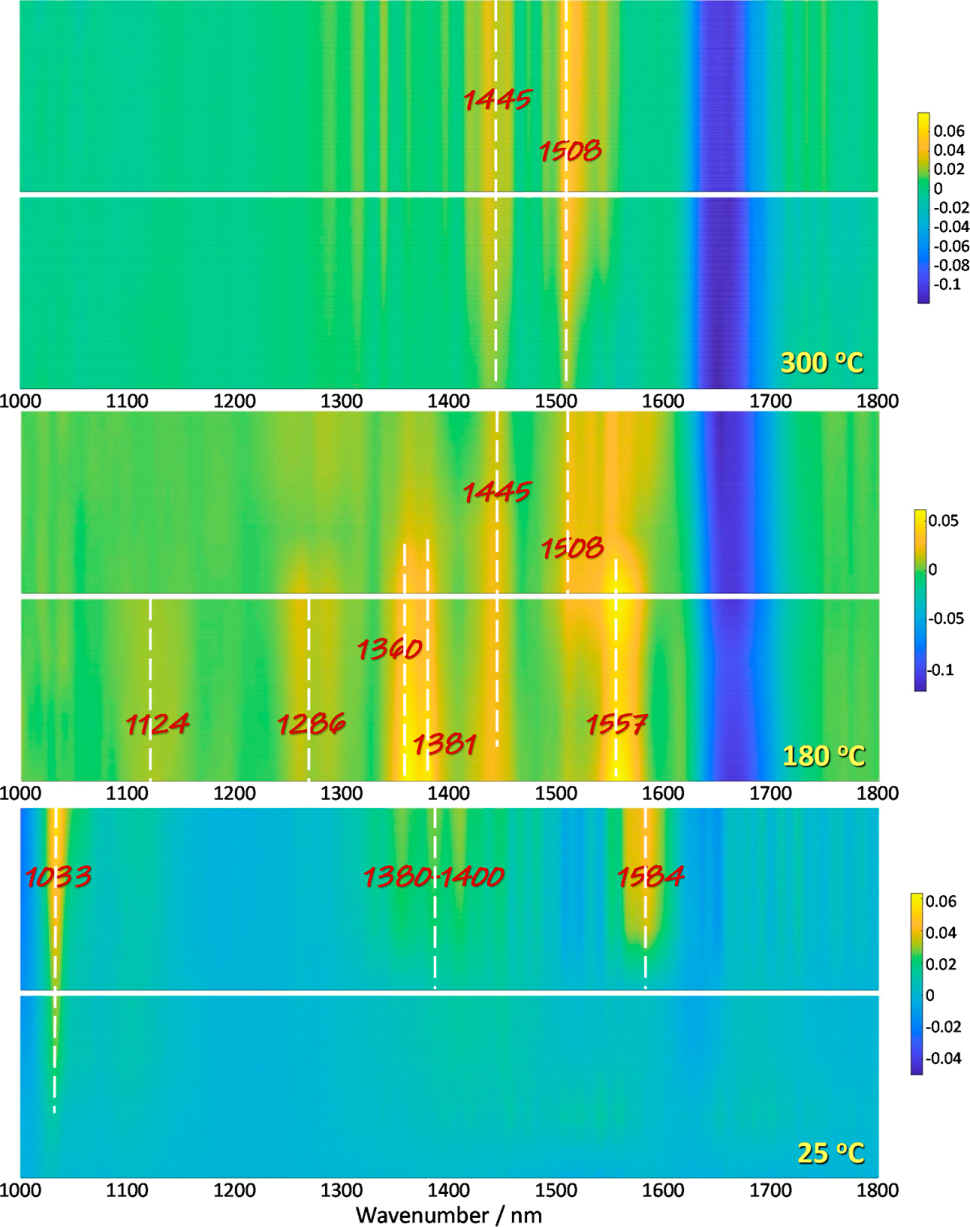
IR spectra of the 0.5Pd/NbTi sample under dark and illuminated conditions at several temperatures. Dark and illuminated results are presented as the bottom and top panels, respectively, for each temperature.

To analyze the origin of the activity and, particularly, to shed light into the effects of the dual excitation with respect to the single source, we performed an in situ IR analysis of the catalysts under reaction conditions. To this end, we explored the activity under photo-alone, thermal-alone (or dark), and thermo-photo conditions at room temperature, at 180 °C, where the maximum thermo-photo effect is achieved, and, finally, at 300 °C, where maximum thermal-alone activity is reached. The IR signal is recorded as a function of time under the influence of the reaction mixture up to stability, for example, absence of changes in the signal. Figures 6 and S5 show results for the 0.5Pd/NbTi and 2.5Pd/NbTi samples, respectively. The fist catalyst maximizes the thermo-photo synergy, while the second is the most active thermo-alone catalyst. As shown in Figure S6, we also provide results concerning the 0.5Pd/Ti reference system to facilitate the analysis of the catalytic effects of Nb on the reaction.

Focusing on the most interesting 0.5Pd/NbTi case, the IR signal at room temperature presents a relatively small number of peaks (Figure 6). Under dark conditions, the evolution of the IR signal with time displays a single signal at ca. 1033 cm^–1^, ascribable to the C–O stretching mode of adsorbed methanol species.^51−53^ Other bands develop significant intensity under illumination. Broad bands at ca. 1584 and 1380–1400 cm^–1^ can be ascribed to carboxylate and carbonate species, with prominent presence of bridged-bonded formate.^30,52,53^ Under thermal-alone conditions at 180 °C, we observe the presence of better defined bands located at ca. 1557 [ν_as_(OCO)], 1381 [δ(C–H)] and 1360 [ν_s_(OCO)] cm^–1^, ascribed to carboxylate-type species. Additional bands at 1286 [coupling of ν(CO) and δ(C–H)) and 1124 (*ν*(C–OH)] cm^–1^ provide evidence of the formation of unperturbed formic acid.^31,53,54^ In addition, a band at 1445 cm^–1^ is observed. Most of the mentioned bands quickly disappear under thermo-photo conditions, leaving a couple of bands at ca. 1508 and 1445 cm^–1^ which would indicate the presence of bridge-bonded and other, like mono-coordinated, carboxylates.^52−54^ These two last bands are also observed at higher reaction temperatures in Figure 6. In this figure, the desorption of surface water species at temperatures above room temperature becomes evident by the negative broad band centered at ca. 1645 cm^–1^, ascribed to the bending mode of the molecule.^53^ Summarizing, the methanol interaction with the catalyst is observed from room temperature, being promoted under illumination, and its surface coverage quickly decreases with temperature, making the signal undetectable at or above 180 °C. At this temperature, carboxylate-type species dominates the surface. Among them, formic acid-type surface species would appear pre-eminent and seem to disappear from the surface under illumination. Other species, like bridged-bonded carboxylate species, mostly remains at the surface.

Comparison with the 2.5Pd/NbTi sample (IR results presented in Figure S5) provides evidence of the similar behavior under dark/illuminated conditions at room temperature. The detection of the same surface species than the ones detected in the 0.5Pd/NbTi sample is evident. However, a modest shift to lower wavenumbers of the carboxylate-type bands at 1580, 1508, 1367, 1288, and 1257 cm^–1^ is observed at this temperature as well as higher reaction temperatures (Figure S5). These bands are however not sensitive to illumination conditions at reaction temperatures equal or above at 180 °C. This main difference between these two (0.5Pd/NbTi and 2.5Pd/NbTi) samples provides evidence that, at the temperature corresponding to the highest thermo-photo effect, the most active sample is able to facilitate the evolution of the formic acid-type surface species, leading to carbon oxides and hydrogen. The presence of Nb is critical to facilitate this step. The 0.5Nb/Ti reference sample does not show evidence of such formic acid species and evolution under the reaction conditions (Figure S6).

Based on the previous physico-chemical characterization and, particularly, using the IR analysis of the samples presented in Figures 6, S5 and S6, we can delineate the chemical ground of the synergistic utilization of light and heat by the Pd/NbTi catalysts. A first point is that carbon oxides are the only gas-phase carbon-containing molecules obtained as products. At the surface of the active materials, formic acid appears as the main active intermediate. The IR shows, on the other hand, the presence of other oxidized carbon-containing entities (carboxylate-type species) at the surface as spectator species. For all catalysts displaying synergy, a reaction mechanism with the following critical steps can be considered
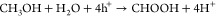
4
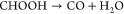
5

**Figure 7:**
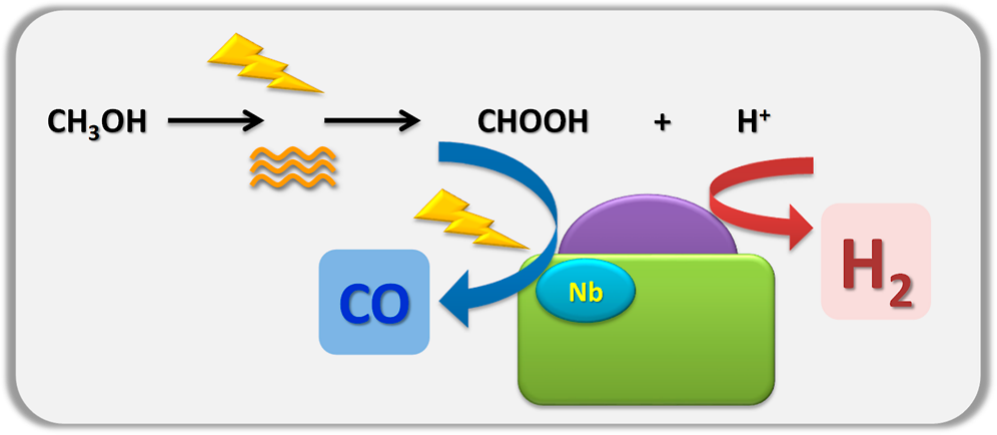
Schematic representation of the Pd–Nb interaction effects on the reaction mechanism.

As well-known and as illustrated in Figure 7, methanol acts as a sacrificial agent of the reaction, and its activation goes through a reaction mechanism using hole-related species as a charge carrier oxidizing agent. Equation 4 summarizes this step-like process which can product several intermediates.^14,21,34,55^ Here, at the surface of the catalyst(s), we only observed the formic acid-type and other (spectator-type) carboxylate species. Together with the production of formic acid-type species, eq 4 shows the concomitant generation of proton entities. The coupling of protons to render hydrogen consumes electrons (2H^+^ + 2e^–^ → H_2_) and, likely, takes place at the noble metal component (Figure 7).^17−19^Equation 5 described the subsequent evolution of the formic acid species at the surface of the active samples. Note that the overall reaction (sum of eqs 4 and 5) can be viewed as a methanol decomposition reaction (CH_3_OH → CO + 2H_2_), but the combined heat and light energy makes a critical influence of the charge carrier species into the mechanism. Also, it is worth to note that water is a necessary reagent in eq 4 but is also produced in eq 5, leading to a null consumption or generation of water and null net contribution to the production of hydrogen. Under dual light-heat conditions, the proposed mechanism highlights the production and activation of formic acid-type surface species as a key point and would lead to the hydrogen to carbon monoxide ratio of 2.

Light effects on the mechanism are explicitly described in eq 4 but, as discussed below, would likely facilitate the reaction presented in eq 5. Note that the presence of Nb near the metal phase and the illumination of the catalyst(s) at all reaction temperatures, but particularly around 180 °C, jointly promote not only the formation but also the evolution of formic acid surface species (Figure 7). Such promotion effects reached the maximum with the 05Pd/NbTi sample. According to eq 5, the formic acid-type surface species suffered a dehydration reaction with the generation of the carbon monoxide component of the syngas mixture.^14,21,34^ For this last step (eq 5), the effect of light could be related to a direct effect (e.g., activation of the molecule by a hole–electron couple) but, according to the IR, to indirect effects originated by altering the surface population of intermediates and, particularly, those located near the formic acid-type species. The latter is suggested by the light-promoting effect on the adsorption of the initial, methanol species, and subsequent change of the surface population of all carbon-containing intermediates. The indirect effect (e.g., through other adsorbates) of light on formic acid dehydration (eq 5) has been previously reported and would be the most probable effect according to our results.^56,57^

To complete the analysis of the reaction mechanism, another important point to analyze is the slightly higher values than 2 of the H_2_/(CO + CO_2_) parameter observed in Figure 2. Greater values are connected to an increasing production of carbon dioxide and CO/(CO + CO_2_) values below 1 in Figure 2. Due to the absence of other than carbon monoxide/dioxide products, this indicates the occurrence of the formic acid dehydrogenation reaction at the catalyst(s) surface, eq 6.
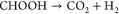
6

Such reaction occurs with production of hydrogen, being an additional source to eq 4. If formic acid dehydrogenation (eq 6) occurs instead of the dehydration process (eq 5), the overall (dehydrogenation) reaction from methanol would be CH_3_OH + H_2_O → CO_2_ + 3H_2_. This explains the values above 2 observed in Figure 2 for the H_2_/(CO + CO_2_) parameter when the simultaneous formation of carbon monoxide and dioxide is detected. Nonetheless, even in the case of prolonged operation (see Figure S3), the contribution of eq 6 and the formation of carbon dioxide is small, about 13% (measured on methanol consumption basis). Besides that, Figure S3 provides evidence that eqs 4–6 account for the majority of the hydrogen produced in our system (ca. 97%). On the other hand, the analysis indicates that eqs 5 and 6 correspond to fast steps of the mechanism. Finally, the practical null contribution of the water gas shift reaction in the case of the Pd/NbTi system can be noticed. This contrast, for example, with other noble metal-promoted systems, and would be directly related to the rather low interaction of carbon monoxide with the noble metal component taking place in our catalysts.^23,31,42^

It should be pointed out that the underlying physico-chemical mechanism allowing heat and light energy handling by the catalytic system is rather complex. In the first (simple) approximation, the thermal energy likely impact in the reagents and intermediates though direct energy absorption (roto-vibrational modes) as well as the energy exchange of the surface moieties with the solid. On the other hand, the UV illumination interacts primarily with the solid and produces the already discussed charge carrier species and subsequent excitation of the molecules located at the catalytic surface.^58,59^ For our system, the strong decay of the activation energy (from the thermal alone result) and the demonstrated synergy (from both thermal and photonic energy points of view) achieved in the chemical process would suggest a different potential energy surface and key carbon-containing intermediates with respect to both single-energy source processes. The thermo-photo process seems therefore unique and different from the parent processes. This fact is evidenced by the IR results and ends up in differences in surface coverage of the adsorbed entities and the key steps of the reaction mechanism related to formic acid formation and evolution. As a result, the study shows that under thermo-photo catalytic conditions, one of our samples, the 0.5Pd/NbTi catalyst, is able to maximize the production of syngas (CO/H_2_) with a synergistic use of the two energy sources of the process.

## Conclusions

In this work, a series of catalysts with growing quantities of Pd deposited on a Nb-doped anatase support was tested for the thermo-photo production of syngas from methanol/water mixtures. The resulting high-surface area (around 120 m^2^ g^–1^) materials contain a pure anatase support with Nb occupying lattice positions of the oxide. Pd is deposited in the 0.1–2.5 wt % percentage in the form of metallic nanoparticles with size going from below 2 nm to ca. 4.5 nm as the Pd content of the series grows. The resulting powders having a Pd content below or equal to 1 wt % are stable under long-term operation (reaction) conditions. Such stability concerns the physico-chemical properties of both the Nb-doped semiconductor as well as the Pd nanoparticles.

The activity of the catalysts was tested in the (thermo-photo) production of syngas under pure photo (room temperature), thermal (also called dark conditions), and photo-thermal (dual excitation) conditions. An impressive performance was evidenced from all perspectives for the catalysts under dual excitation. Values of 1.8% of the energy efficiency if expressed as hydrogen production, as well as overall excess rate and quantum efficiency values exceeding the additive use of light and heat by, respectively, ca. 55 and 65% were obtained for the best 0.5Pd/NbTi catalyst. Moreover, the join analysis of these three parameters, the energy efficiency, the excess reaction rate, and the excess quantum efficiency of the reaction provided conclusive proof that the 0.5Pd/NbTi sample is able to synergistically utilize light and heat under dual excitation. Such synergy leads to a top performing system among the ones presented in the literature. The physico-chemical origin of the catalytic promotion of activity with respect to the single-source situation(s) was examined using PL and in situ IR spectroscopies. The results (summarized in eqs 4–6) showed that the 05Pd/NbTi catalyst promotes the generation and evolution of formic acid-type surface species under dual excitation. Therefore, the production of syngas (eqs 4 and 5) takes place with a catalytic system that can use the heat-light combination in a rather efficient way, providing a ground to improve both thermal and light-related processes and contributing to the opening of new ways for the efficient generation of the mentioned energy vectors from renewable chemicals.

## Supporting Information Available

Experimental details about characterization and catalytic measurements, results showing catalytic activity, TEM-derived primary particle size of the noble metal, and in situ infrared results under reaction conditions (PDF)
