# Supplementary material for: Green Thermo-Photo Catalytic Production of Syngas Using Pd/Nb–TiO2 Catalysts
Source: ACS Sustain Chem Eng. 2023 Feb 20;11(9):3896–906. doi: 10.1021/acssuschemeng.2c07285 (PMC9993398; doi:10.1021/acssuschemeng.2c07285)
Supplement: Supplementary file 1 — sc2c07285_si_001.pdf [file sc2c07285_si_001.pdf]

# Supporting Information

# Green Thermo-Photo Catalytic Production of Syngas using Pd/Nb-$TiO_2$ catalysts

*Uriel Caudillo-Flores,[1] Rocío Sayago,[2] Alejandro Ares-Dorado,[2 2] Sergio Fuentes-Moyado,[1] Marcos Fernández-García,[*2] and Anna Kubacka[*2]*

1) *Centro de Nanociencias y Nanotecnología, Universidad Nacional Autónoma de México, Ensenada 22800, Mexico.*
2) *Instituto de Catálisis y Petroleoquímica, CSIC.C/Marie Curie 2, 28049-Madrid, Spain.*

*Email: A.K. (ak@icp.csic.es), M. F-G (mfg@icp.csic.es)*

Number of Pages: 16

Number of Figures: 6

Number of Tables: 1

## Characterization

Elemental analysis was determined using inductively coupled plasma atomic absorption spectroscopy (ICP-OES) (PerkinElmer Optima 3300 DV). A Seifert D-500 diffractometer equipped with Ni-filtered Cu Kα radiation was employed to record XRD pattern of the as-synthesized samples with a 0.02° step. The particle sizes were estimated using XRD using the Williamson–Hall formalism [1]. UV–vis diffuse-reflectance spectroscopy experiments were performed on a Shimadzu UV2100 apparatus using nylon as a reference and the results presented as Kubelka-Munk transform [2]. Band gap analysis for the titania (anatase) indirect gap semiconductor was done following standard procedures; e.g. plotting $(hva)^n$ (n= ½ or 2 for indirect or direct semiconductor; hv =excitation energy, a=absorption coefficient) vs. energy and obtaining the corresponding intersection of the linear fit with the baseline [3]. Transmission electron microscopy images were taken with a JEOL 2100F TEM/STEM microscope. UV−vis transmission or diffuse-reflectance spectra were recorded with a Shimadzu UV2100 apparatus (using $BaSO_4$ or Teflon as a reference for diffuse experiments). XPS data were recorded on 4 × 4 $mm^2$ pellets, 0.5 mm thick, prepared by slightly pressing the powered materials, which were outgassed in the prechamber of the instrument at room temperature up to a pressure < $2 \times 10^{-5}$ Pa to remove chemisorbed water from their surfaces. The XPS spectra of the samples were recorded using a SPECS® spectrometer with a PHOIBOS® 150 WAL hemispherical energy analyzer with angular resolution (<0.5 degrees), equipped with an XR 50 Al-X-ray and μ-FOCUS 500 X-ray monochromator (Alexcitation line) sources. Samples were first degassed at

$10^{-5}$ mbar in the pretreatment chamber before being transferred to the analysis chamber, where residual pressure was kept below $5x10^{-9}$ mbar during data acquisition. The binding energies (BE) were referenced to the C 1s peak (284.8 eV) to account for the charging effects. Photoluminescence spectra were measured at room temperature on a Fluorescence Spectrophotometer (Perkin Elmer LS50B).

Diffuse Reflectance Infrared Fourier Transform Spectra (DRIFTS) were taken in a Bruker Vertex 80 FTIR spectrometer using a MCT detector and running under OPUS/IR software. The set-up consists of a praying mantis DRIFTS accessory (Harrick Scientific) and a reaction cell (HVC, Harrick Scientific). The reaction mixture was prepared by injecting in a nitrogen carrier (10 mL $min^{-1}$) a 3:7 alcohol:water mixture (0.15 mL $min^{-1}$) with a syringe pump before entering the DRIFTS cell. This mirrored the conditions at the reactor (see below). The DRIFTS spectra were collected in the range of 4000–600 $cm^{-1}$ with a resolution of 4 $cm^{-1}$. In DRIFTS experiments in-situ light excitation was carried out using 365 nm radiation. A Hg-Xe 500 W lamp with a dicroic filter 280-400 nm coupled with a 365 nm (25 nm half-width) filter (LOT-Oriel) were used to select the light excitation. Each sample, without any previous treatment (expect flowing nitrogen for 10 min), was subjected in a continuous mode (without modifying gas mixture) to an experiment which aims to test the: *i)* dark (thermal-alone) conditions, *ii)* following the evolution under illumination conditions at the desired temperature (thermo-photo experiments). Experiments are carried out at room temperature (RT), 180 and 240 °C. Spectra are presented as the difference of the spectrum taken at the initial one (t=0) of the whole treatment.

## Catalytic set-up and details

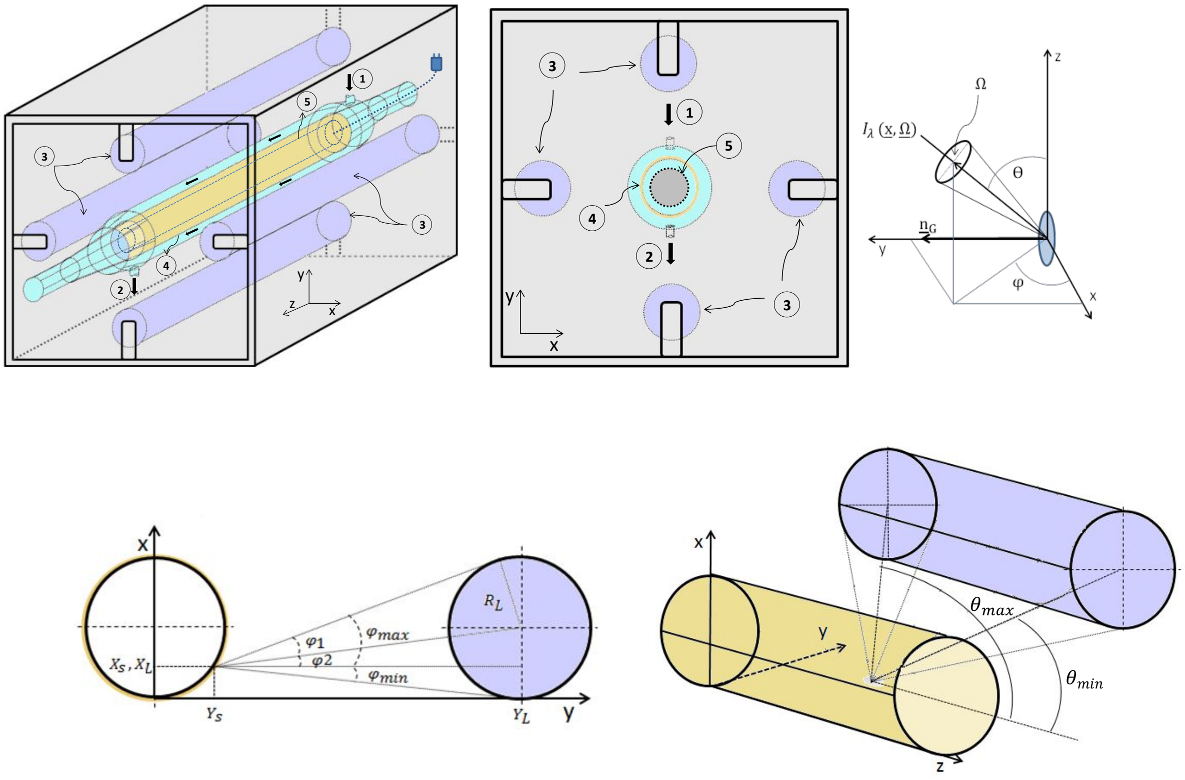


Figure S1. Upper, Left and Center: Photocatalytic annular reactor scheme; side and front views: (1) Gas inlet, (2) gas outlet, (3) UV lamp, (4) catalyst (brown) sample, (5) cartridge heater. $q_{sup}$ Radiation flow on the surface of the sample (red), $q_n$ radiation flow from the lamps (blue). Upper, Right: Center of coordinates located at the sample (defined by coordinates $x_s$, $y_s$, $z_s$). Down, Coordinate system to define the integration limits of radiation Model. (Left) $\varphi_{min}$ and $\varphi_{max}$. (Right) $\Theta_{min}$ and $\Theta_{max}$.

The thermo-photo activity of the samples for liquid-phase methanol reforming was tested using a gas-phase continuous flow annular thermo-photo-reactor (pyrex) schematically depicted in

Figure S1. The catalyst (ca. 0.25 mg $cm^{-2}$) was deposited onto the inner tube as a thin layer from a suspension in ethanol. During thermo-catalytic and thermo-photo-catalytic tests, the film was heated using a cartridge heater. The temperature of the layer was controlled and monitored by a temperature controller (Toho TTM-005) and K-type thermocouple inserted into the reactor. Minimal (below 1 °C) axial temperature variation was reached with a cartridge heater (230 V; 500 W; "Resistencias RSI INCOLOID800") having controlled/compensated homogeneous heating. The UV irradiation was generated by four fluorescent UV lamps (Philips TL 6 W/08-F6T5 BLB, 6 W) symmetrically positioned outside the reactor. For visible light 6 W, Sylvania F6 W/ lamps were used with appropriate filters (LOT Quantum). The lamps provide ca. 8 (UV) or 12 mW $cm^{-2}$ (visible) intensity at the sample surface. Full details about energy distribution and illumination intensity can be obtained from previous publications [4]. The reacting mixture 30:70 $CH_3OH$:$H_2O$ (0.15 mL $min^{-1}$) was injected with a syringe pump in an Argon carrier flow (10 mL $min^{-1}$). The methanol to water ratio has been shown to maximize hydrogen production in noble metal based anatase catalysts [5,6,7]. The gas line was also heated in order to minimize liquefaction of the methanol:water mixture. The catalytic properties were evaluated at 3 h from the start of the irradiation, where a pseudo-stationary situation is reached. The concentrations of

the reaction products were analyzed using an online mass spectra (Onmistart 300) and gas chromatograph (Agilent GC 6890) equipped with HP-PLOT-Q/HP-Innowax columns (0.5/0.32 mm I.D. × 30 m) as well as thermal conductivity and flame ionization detectors. Catalytic tests were repeated 3 to 4 times and error values calculated from the corresponding set of data.

In this work we measured catalytic output with the help of three observables, the reaction rate, the quantum efficiency and the global energy balance of the reaction. The reaction rate (r) measured the number of hydrogen production molecules per surface area and time unit, but to analyze the thermo-photo production of hydrogen we defined an "excess rate" ($r_e$) measured trough equation S1.

$$r_e = r_{\text{(Thermo-photo)}} - (r_{\text{(Photo)}} + r_{\text{(Thermo)}}) \qquad \text{(S1)}$$

Such "excess" rate measured the potential synergy occurring between both energy sources in the thermo-photo catalytic process. Synergy is thus measured as the excess (i.e. positive value) over the additive effect of light and heat in the reaction rate.

The second is the Quantum Efficiency (QE) parameter for hydrogen production. QE is defined by Equation S2 [8].

$$QE(\%) = 100 \times \frac{2 \times r\ (\text{mol m}^{-2}\text{s}^{-1})}{e^{a,s}\ (\text{Einstein m}^{-2}\text{s}^{-1})} \qquad \text{(S2)}$$

In this equation, r is the reaction rate and $e^{a,s}$ the average local superficial rate of photon absorption. The factor two consider the requirement of two electrons per hydrogen molecule. Here, for the calculation of the quantum efficiency, we will use two different reaction rates, the normal ones and the excess one. The use of the latter would allow to measure an "excess" quantum efficiency.

The rate of hydrogen production is measured using mass spectrometry and gas chromatography as previously outlined and normalized using the BET surface area of the sample. The local superficial rate of photon absorption ($e^{a,s}$) is defined by Equation S3. It follows from the equation corresponding to a pure photo-catalytic process but eliminating the losses coming from charge emission with temperature [4]. In this equation $F_{A_S}$ is the fraction of light absorbed by the sample $q_{sup}$ the radiation flux at each position ($\underline{x} \equiv X_s, Y_s, Z_s$) of the catalytic film, and $T_e$ is the thermal emission loss terminus.

$$e^{a,s}(\underline{x}) = \left(q_{sup}(\underline{x}) - T_e\right) F_{As} \qquad \text{(S3)}$$

To obtain the radiation flow on the surface of the samples, we calculate first the impinging radiation flux from the lamps ($q_n$). Considering the coordinate system presented in Figure S1 and the geometry of the reactor (annular multilamp), the $q_n$ can be determined by Equation S4 [4].

$$q_{\mathrm{n}}\left(X_s, Y_s, Z_s\right) = \sum_{L=1}^{L=4} \sum_{\lambda} \int_{\varphi_{min,L(\mathrm{x,y})}}^{\varphi_{\max,L\,(\mathrm{x,y})}} \int_{\Theta_{min(\mathrm{x,y},\varphi)}}^{\Theta_{max(\mathrm{x,y},\varphi)}} \frac{P_{\lambda,L}}{2\pi\, R_L\, Z_L} \sin^2\Theta \; \left(\left(\frac{X_s - X_L}{R}\right) \cos\varphi + \left(\frac{y_s}{R}\right) \sin\varphi\right) d\varphi d\Theta \qquad \text{(S4)}$$

Where $Xs$, $Ys$, $Zs$ are the coordinates of the points located on the surface of the catalytic films, and $X_L$, $Y_L$, $Z_L$ which are the coordinates of the points located on the surface of the lamp. R type variables correspond to the radius of the cylinder supporting the sample ($R$) or of the lamp ($R_L$), see Figure S1. Angular variables (Θ,φ) are defined as described in Figure S1. Integration limits of equation S4 are summarized in equations S5-S12 and can be graphically visualized in Figure S1.

$$\varphi_1 = \tan^{-1}\left(\frac{X_L - Xs}{Y_L - Ys}\right) \qquad \text{(S5)}$$

$$\varphi_2 = \sin^{-1}\left(\frac{R_L}{(X_L - X_s)^2 + (Y_L - Y_s)^2}\right) \qquad \text{(S6)}$$

$$\varphi_{min} = \varphi_1 - \varphi_2 \qquad \text{(S7)}$$

$$\varphi_{max} = \varphi_1 + \varphi_2 \qquad \text{(S8)}$$

$$\Theta_{min}(\varphi) = \cos^{-1}\frac{-Z_s}{(X_{Lm}(\varphi) - X_s)^2 + (Y_{Lm}(\varphi) - Y_s)^2 + Z_s{}^2} \qquad \text{(S9)}$$

$$\Theta_{max}(\varphi) = \cos^{-1}\frac{Z_L - Z_s}{(X_{Lm}(\varphi) - X_s)^2 + (Y_{Lm}(\varphi) - Y_s)^2 + Z_s{}^2} \qquad \text{(S10)}$$

Where:

$$X_{Lm}(\varphi) = X_L + (X_s - Y_L)\cos\varphi^2 + (Y_L - Y_s)(\cos\varphi \sin\varphi) - \sin\varphi \sqrt{(R_L{}^2 - (X_s - X_L)\cos\varphi + (Y_L - Y_s)\sin\varphi)^2)}$$

(S11)

$$Y_{Lm}(\varphi) = Ys_i + (Y_L - Y_s)\cos\varphi^2 + (X_s - X_L)(\cos\varphi \sin\varphi) - \cos\varphi \sqrt{(R_L{}^2 - (X_s - X_L)\cos\varphi + (Y_L - Y_s)\sin\varphi)^2)}$$

(S12)

Where symbols $Xs$, $Ys$, $Zs$ and R are the coordinates of the points located on the surface of the catalytic films and the radius of the cylinder supporting the sample , and $X_L$, $Y_L$, $Z_L$ which are the coordinates of the points located on the surface of the lamp. Finally, the $q_{sup}$ x/y components (see Figure S1; Equation S13) can be determined using $q_n$ and a radiation balance, which considers the main optical (Transmittance, $F_i$, and Reflectance, $R_i$) events occurring in all components of the reactor placed between the emission source and catalyst, i.e. glass and reaction media, as well on the catalytic film.

$q_{sup}^{x,y} = f\,(q_n, F_i, R_i)$;i = catalyst, glass, reaction media (S13)

A detailed description of the mathematical formulation to provide $q_{sup}$ as a function of $q_n$ (Equation S13) and the transmittance/reflectance optical measurements for each component of our reactor system can be found elsewhere [9].

The $T_e$ is a loss term  and can be calculated using equation S13 is calculated using considering that the emission of a body in a medium can be calculated using the Plank´s law [10]. The radiation intensity per surface area unit is [11]:

$$T_e = \frac{n^2 \pi\, h\, T^4}{c^2} \int_0^\infty \frac{\gamma^3\, \alpha(\gamma,T)}{e^{\frac{h\gamma}{k\,T}} - 1}\, d\gamma \qquad \text{(S13)}$$

Where $\gamma$ is the photon frequency, h is the Plank-s constant, c is the speed of light, n is the refraction index of the solid, k is the Boltzmann´s constant, T is the temperature of the sample and $\alpha$ is the absorption efficiency that acts as an emissivity type factor as discussed in refs. 4,10. This $T_e$ term is negligible at the temperatures of this work as it only makes a maximum correction of 4 parts per million to the local superficial rate of photon absorption values. This is at least 3-4 orders of magnitude below the standard error of the $e^{a,s}$ coefficient. Such a result is somehow expected as emission losses in titania-based (the dominant component) materials are known to occur at higher temperatures than here used [12].

Finally, an energy balance of the thermo-photo process is carried out to compare with the simple sum of the thermal and photo processes. Taking the solar to hydrogen parameter as a guide [13], the energy enclosed in the hydrogen products is calculated as the ratio between the enthalpy of the hydrogen burning reaction (+23.7 kJ $mol^{-1}$) and the energy required to produce it using the (simple sum of the energy consumed by) thermal and photon sources described previously.

## Catalytic and Characterization Results

Table S1. Hydrogen reaction rate and quantum efficiency obtained under photo, thermo and thermo-photo excitation using the 0.5Pd/NbTi catalyst.

| **Parameter** | **Activity at RT (Photo) or 180 ºC (Thermo and Thermo-Photo)** | | | | |
|---|---|---|---|---|---|
| | **Photo** | | **Thermo** | **Thermo-photo** | |
| | UV | Visible | | UV | Visible |
| **Rate (mmol $g^{-1}$ $h^{-1}$)** | $3.8_5$ | 0.36 | 2.1 | 13.0 | $3.2_5$ |
| **QE (%)** | 2.4 | 0.1 | - | 8.2 | 2.6 |

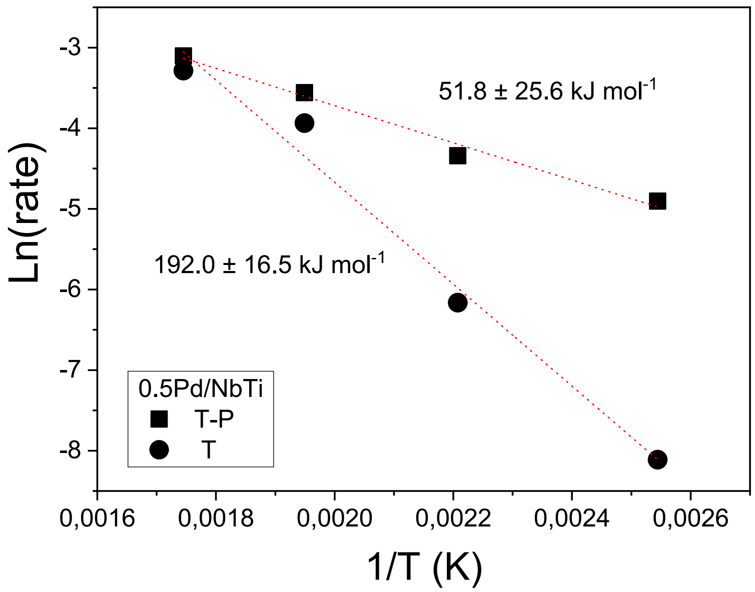


Figure S2. Arrhenius plot for the 0.5Pd/NbTi sample under single thermal (T) and dual thermo-photo (T-P) excitation.

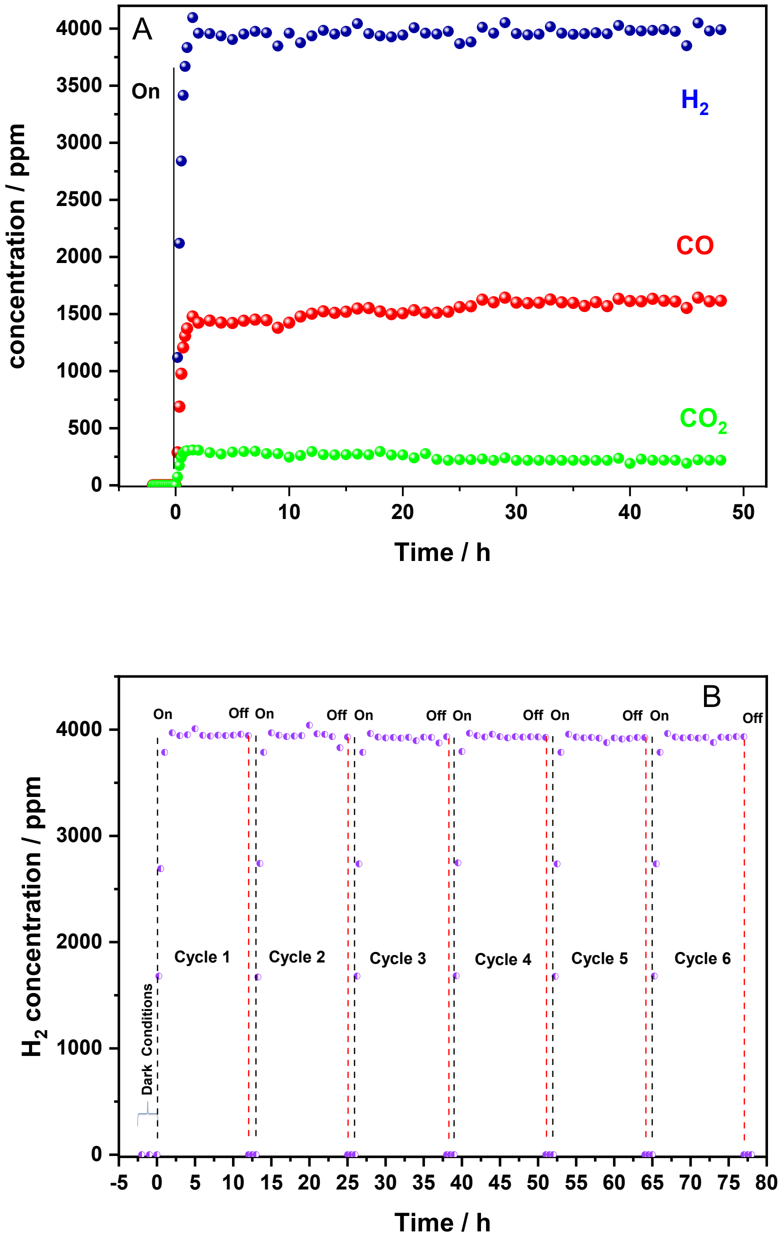


Figure S3. Long term activity tests for methanol photo-reforming using the 0.5Pd/NbTi sample. Temperature 180 °C. (A) Continuous run for ca 50 h. (B) 6 consecutive cycles of ca. 10

## Characterization Results

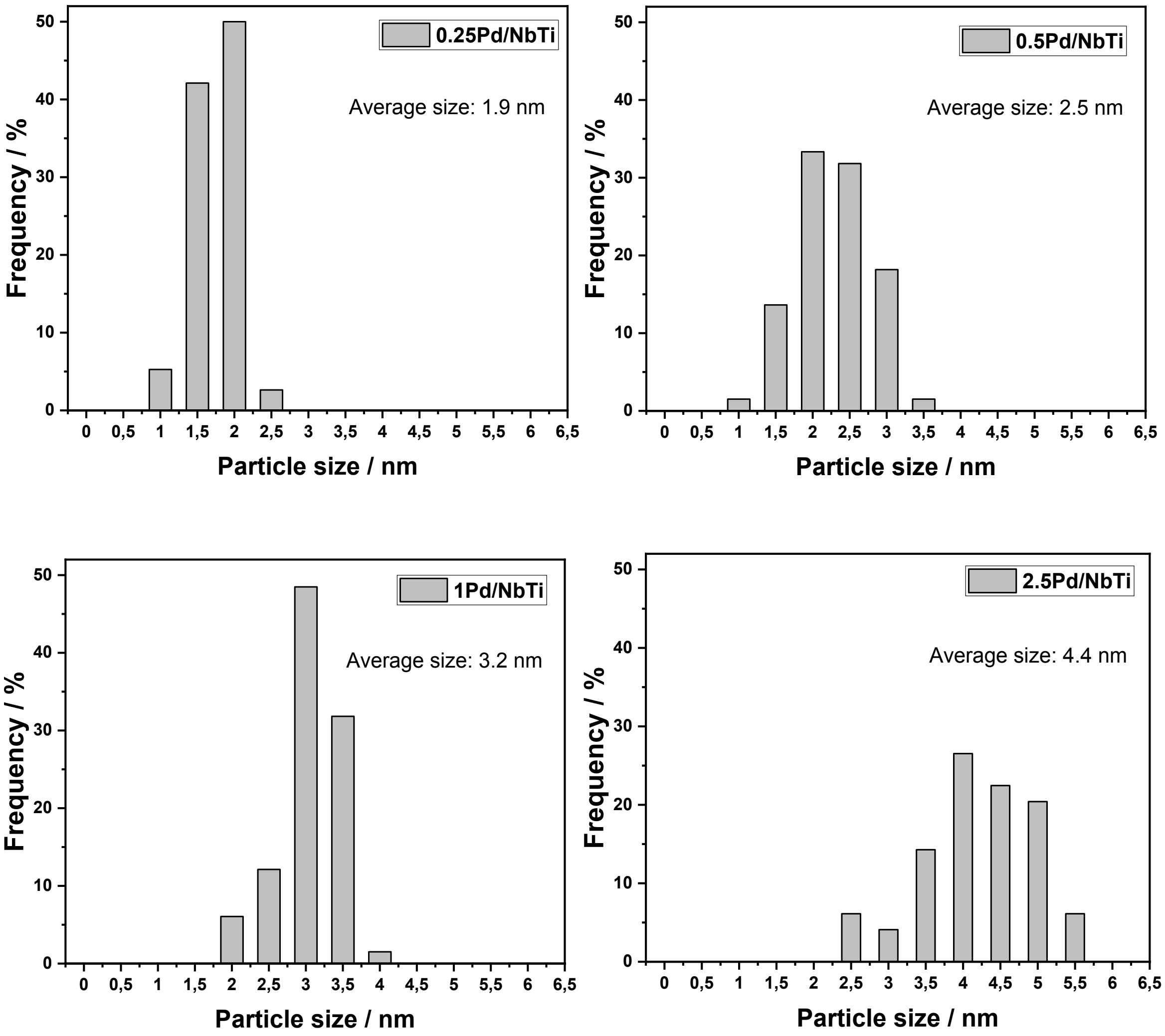


Figure S4. Pd particle size distribution obtained from TEM measurements. (A) 0.25Pd/NbTi, (B) 0.5Pd/Nb/Ti, (C) 1Pd/NbTi, and (D) 2.5Pd/NbT samples.

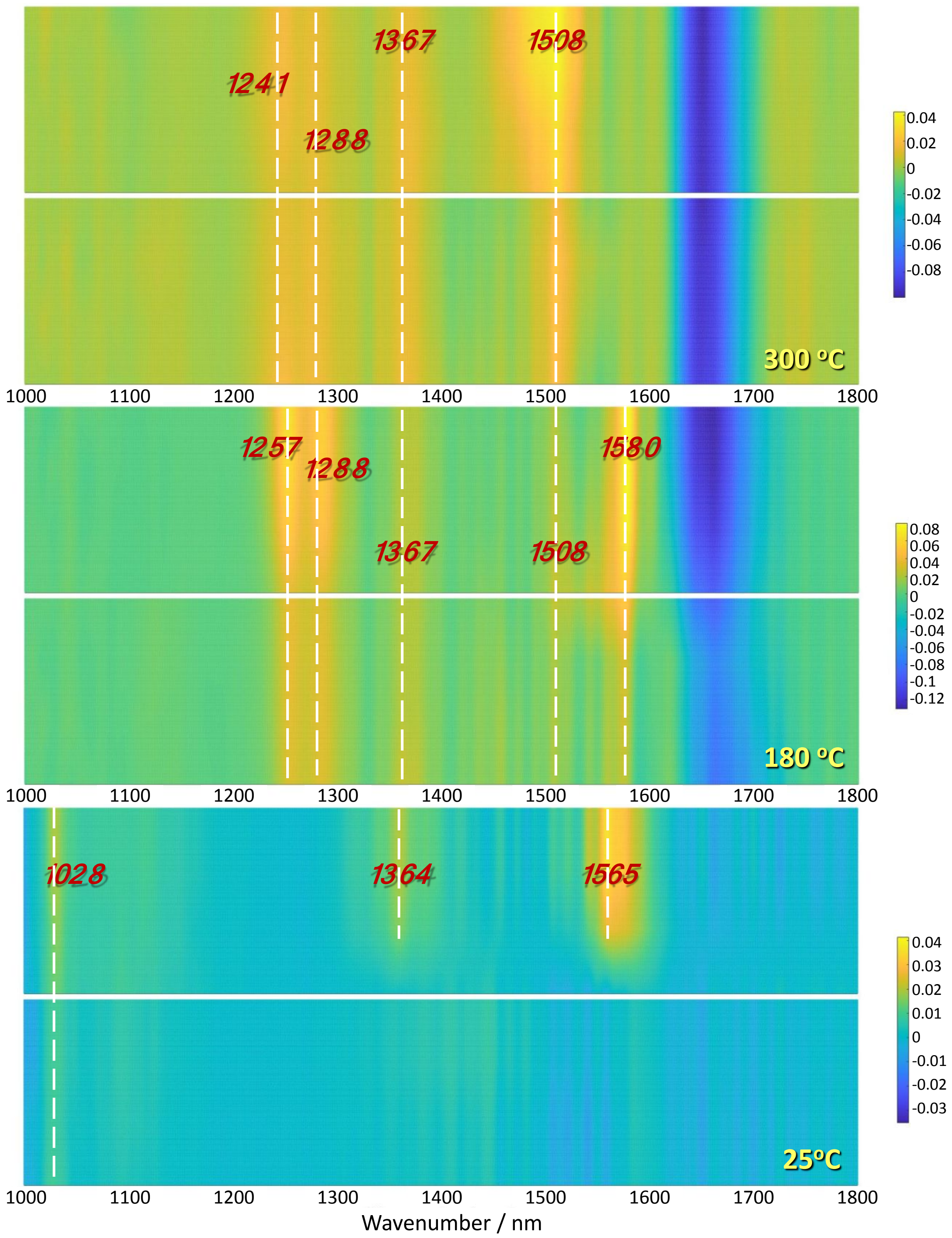


Figure S5. IR spectra of the 2.5Pd/NbTi sample under dark and illuminated conditions at several temperatures. Dark and illuminated results are presented as the bottom and top panels, respectively, for each temperature

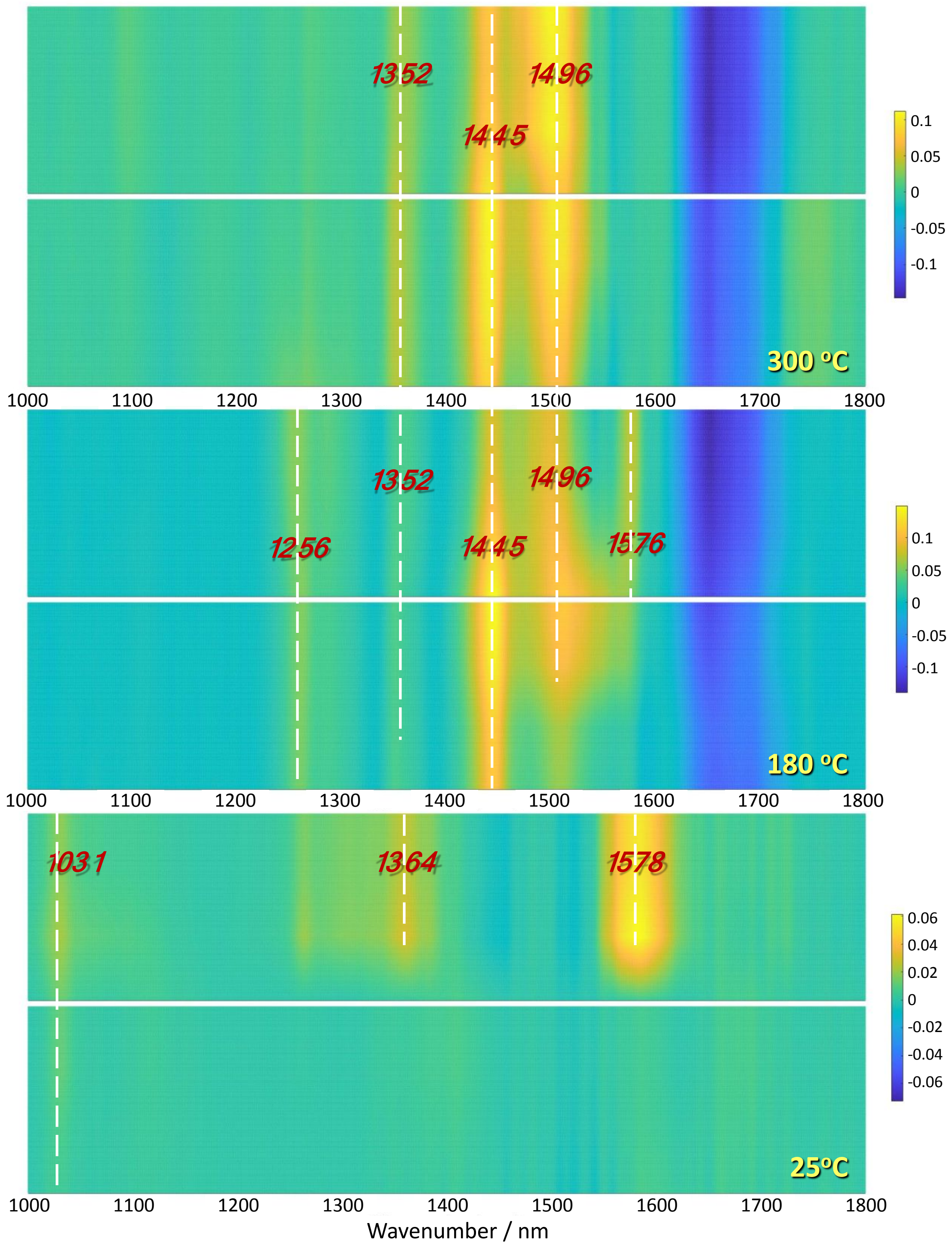


Figure S6. IR spectra of the 0.5Pd/Ti reference sample under dark and illuminated conditions at several temperatures. Dark and illuminated results are presented as the bottom and top panels, respectively, for each temperature.

**References**


1 G.K. Willianson, W.H. May. X-ray line broadening from filed aluminium and wolfram. Acta Metall. 1 (1953) 22-29. https://doi.org/10.1016/0001-6160(53)90006-6

2 P. Kubelka. New Contributions to the Optics of Intensely Light-Scattering Materials. Part I. J. Op. Soc. Am. 38 (1948) 448-457. https://doi.org/10.1364/JOSA.38.000448

3 M. Fernández-García, A. Martínez-Arias, J.C. Hanson J.A. Rodriguez. Nanostructured Oxides in Chemistry: Characterization and Properties. Chem. Rev. 104 (2004) 4063-4105. https://doi.org/10.1021/cr030032f

4 M.J. Muñoz-Batista, A. Kubacka, A.B. Hungría, M. Fernández-García. Heterogeneous photocatalysis: Light-matter interaction and chemical effects in quantum efficiency calculations. J. Catal. 330 (2015) 154–166. https://doi.org/10.1016/j.jcat.2015.06.021

5 G.L. Chiarello, M.H. Aguirre, E. Selli. Hydrogen production by photocatalytic steam reforming of methanol on noble metal-modified TiO2. J. Catal. 273 (2010) 182–190. https://doi.org/10.1016/j.jcat.2010.05.012

6 W.-T. Chen, A. Chan, D. Sun-Waterhouse, J. Llorca, H. Idriss, G.N. Waterhouse. Performance comparison of Ni/TiO2 and Au/TiO2 photocatalysts for H2 production in different alcohol-water mixtures. J. Catal. 367 (2018) 27-42. https://doi.org/10.1016/j.jcat.2018.08.015

7 U. Caudillo-Flores, M.J. Muñoz-Batista, A. Kubacka, M. Fernández-García. Bimetallic Pt-Pd co-catalyst Nb-doped $TiO_2$ materials for $H_2$ photo-production under UV and Visible light illumination. Appl. Catal. B 238 (2018) 533–545. https://doi.org/10.1016/j.apcatb.2018.07.047

8 S.E. Braslavsky, A.M. Braun, A.E. Cassano, A.V. Emeline, M.I. Litter, L. Palmisano, V.N. Parmon, N. Serpone. Glossary of terms used in photocatalysis and radiation catalysis (IUPAC Recommendations 2011). Pure Appl. Chem. 83 (2011) 931-1014. http://dx.doi.org/10.1351/PAC-REC-09-09-36

9 G.E. Imoberdorf, H.A. Irazoqui, A.E. Cassano, O.M. Alfano. Photocatalytic Degradation of Tetrachloroethylene in Gas Phase on TiO2 Films: A Kinetic Study. Ind. Eng. Chem. Res. 44 (2005) 6075–6085. https://doi.org/10.1021/ie049185z

10 G.W. Kattawar, M. Eisner, Radiation from a homogeneous isothermal sphere. Appl. Optics 9 (1970) 2685-90.

11 C.F. Bohm, D.R. Hoffman, Absorption and scattering of light by small particles. Whiley (1999) New York.

12 J. Xhen, P. Vincent, N.P. Blanchard, J. Nicolle, M. Choueib, V. Salles, A. Brioude. Physical properties of individual anatase TiO2 nanowires investigated by field emission in a transmission electron microscope. J. Vac. Sci. Technol. 30 (2012) 011801. https://doi.org/10.1116/1.3668121

13 T. Hisatomi, K. Domen. Reaction systems for solar hydrogen production via water splitting with particulate semiconductor photocatalysts. Nat. Catal. 2 (2020) 387-399. https://doi.org/10.1038/s41929-019-0242-6
